# Pediatric patients’ reasons for visiting dentists in all WHO regions

**DOI:** 10.1186/s12955-021-01801-0

**Published:** 2021-06-13

**Authors:** Katrin Bekes, Mike T. John, Ksenija Rener-Sitar, Mohammad H. Al-Harthy, Ambra Michelotti, Daniel R. Reissmann, Julijana Nikolovska, Sahityaveera Sanivarapu, Folake B. Lawal, Thomas List, Sanja Peršić Kiršić, Ljiljana Strajnić, Rodrigo Casassus, Kazuyoshi Baba, Martin Schimmel, Ama Amuasi, Ruwan D. Jayasinghe, Sanela Strujić-Porović, Christopher C. Peck, Han Xie, Karina Haugaard Bendixen, Miguel Angel Simancas-Pallares, Eka Perez-Franco, Mohammad Mehdi Naghibi Sistani, Patricia Valerio, Natalia Letunova, Nazik Nurelhuda, David W. Bartlett, Ikeoluwa A. Oluwafemi, Saloua Dghoughi, Joao N. A. R. Ferreira, Pathamas Chantaracherd, Stella Sekulić

**Affiliations:** 1 Department of Pediatric Dentistry, University Clinic of Dentistry, Medical University of Vienna, Vienna, Austria; 2 Department of Diagnostic and Biological Sciences, School of Dentistry, University of Minnesota, Minneapolis, MN USA; 3 Department of Prosthodontics, Faculty of Medicine, University of Ljubljana, Ljubljana, Slovenia; 4 Department of Prosthodontics, University Dental Clinics, University Medical Center Ljubljana, Ljubljana, Slovenia; 5 Department of Oral Basic & Clinical Sciences, Faculty of Dentistry, Umm Al-Qura University, Makkah, Saudi Arabia; 6 Section of Orthodontics, Department of Neurosciences, University of Naples “Federico II”, Naples, Italy; 7 Department of Prosthetic Dentistry, University Medical Center Hamburg – Eppendorf, Hamburg, Germany; 8 Department for Prosthodontics, Faculty of Dental Medicine, University Ss. Cyril and Methodius, Skopje, Macedonia; 9 Department of Periodontics, Care Dental College, Guntur, Andhra Pradesh India; 10 Department of Periodontology and Community Dentistry, University of Ibadan and University College Hospital, Ibadan, Nigeria; 11 Department of Orofacial Pain and Jaw Function, Faculty of Odontology, Malmö University, Malmö, Sweden; 12 Department of Prosthodontics, School of Dental Medicine, University of Zagreb, Zagreb, Croatia; 13 Clinic for Dentistry of Vojvodina, Faculty of Medicine, University of Novi Sad, Novi Sad, Serbia; 14 Department of Orofacial Pain, Faculty of Medicine, University of Desarrollo, Santiago, Chile; 15 Department of Prosthodontics, Showa University Dental Hospital, Showa University, Tokyo, Japan; 16 Division of Gerodontology, Department of Reconstructive Dentistry and Gerodontology, University of Bern, Bern, Switzerland; 17 Division of Gerodontology and Removable Prosthodontics, University Clinics of Dental Medicine, University of Geneva, Geneva, Switzerland; 18 Department of Child Health and Orthodontics, Kwame Nkrumah University of Science and Technology, Kumasi, Ghana; 19 Department of Oral Medicine and Periodontology, Faculty of Dental Sciences, University of Peradeniya, Peradeniya, Sri Lanka; 20 Department of Prosthodontics, Faculty of Dentistry with Clinics, University of Sarajevo, Sarajevo, Bosnia and Herzegovina; 21 Sydney Dental School, Faculty of Medicine and Health, University of Sydney, Sydney, Australia; 22 Department of Stomatology, Huashan Hospital, Fudan University, Shanghai, China; 23 Department of Dentistry and Oral Health, Aarhus University, Aarhus, Denmark; 24 Division of Pediatric and Public Health, Adams School of Dentistry, University of North Carolina at Chapel Hill, Chapel Hill, NC USA; 25 Center for Headaches, Facial Pain and TMD, Punta Pacifica Medical Center, Panama City, Panama; 26 Oral Health Research Center, Health Research Institute, Babol University of Medical Sciences, Babol, IR Iran; 27 Instituto Patricia Valério, Belo Horizonte, Minas Gerais Brazil; 28 Department of Anesthesia in Dentistry, Moscow State University of Medicine and Dentistry, Moscow, Russia; 29 Faculty of Dentistry, University of Khartoum, Khartoum, Sudan; 30 Department of Prosthodontics, Faculty of Dentistry, Oral & Craniofacial Sciences, King’s College London, London, UK; 31 Department of Oral Medicine and Periodontology, University of the Western Cape, Cape Town, South Africa; 32 Oral Surgery Department, Faculty of Dentistry of Rabat, Mohammed V University in Rabat, Rabat, Morocco; 33 Exocrine Gland Biology and Regeneration Research Group, Faculty of Dentistry, Chulalongkorn University, Bangkok, Thailand; 34 Faculty of Dentistry, National University of Singapore, Singapore, Singapore; 35 Department of Diagnostic and Biological Sciences, Faculty of Dentistry, Western University, Bangkok, Thailand

**Keywords:** Oral health, Oral health-related quality of life, Surveys and questionnaires, Dentistry, Child, WHO

## Abstract

**Background:**

Oral Function, Orofacial Pain, Orofacial Appearance, and Psychosocial Impact are the four oral health-related quality of life (OHRQoL) dimensions (4D) or areas in which oral disorders impact pediatric patients. Using their dentists' assessment, the study aimed to evaluate whether pediatric dental patients' oral health concerns fit into the 4D of the Oral Health-Related Quality of Life (OHRQoL) construct.

**Methods:**

Dentists who treat children from 32 countries and all WHO regions were selected from a web-based survey of 1580 international dentists. Dentists were asked if their pediatric patients with current or future oral health concerns fit into the 4D of the Oral Health-Related Quality of Life (OHRQoL) construct. Proportions of all pediatric patients’ oral health problems and prevention needs were computed.

**Findings:**

Data from 101 dentists treating children only and 523 dentists treating children and adults were included. For 90% of pediatric patients, their current oral health problems fit well in the four OHRQoL dimensions. For 91% of oral health problems they intended to prevent in the future were related to these dimensions as well. Both numbers increased to at least 96% when experts analyzed dentists´ explanations of why some oral health problems would not fit these four categories.

**Conclusions:**

The study revealed the four fundamental components of dental patients, i.e., the four OHRQoL dimensions (Oral Function, Orofacial Pain, Orofacial Appearance, and Psychosocial Impact) are also applicable for pediatric patients, regardless of whether they have current or future oral health concerns, and should be considered when measuring OHRQoL in the pediatric dental patient population.

**Supplementary Information:**

The online version contains supplementary material available at 10.1186/s12955-021-01801-0.

## Background

Pediatric dental patients visit dentists due to two main concerns—their parents or guardians have noticed oral health problems in their children, or they want to prevent them in the future. Although there has been a general improvement in children’s oral health over the last decades in all WHO regions, dental problems remain highly prevalent during childhood [1]. Among several oral disorders, dental caries is the most prevalent diseases in pediatric dental patients across the globe [2]. Early Childhood Caries is a global public health problem leading to medical, social, and economic consequences [3]. Besides caries, other dental problems such as periodontal disease, malocclusions, and traumatic teeth injuries are frequent oral concerns of the pediatric dental patient and family [4].

The concept of oral health-related quality of life (OHRQoL) has become an important measure to assess biological, social, psychological, and cultural factors related to oral health in children and adults because clinical indicators alone do not fully reveal the impact of oral conditions on general health [5]. Interest has grown enormously in the assessment of OHRQoL among pediatric dental patients in recent years. At present, it is well known that orofacial disorders in general, have an impact on physical functioning and psychosocial well-being on pediatric dental patients and their families, causing pain and discomfort [6]. However, specific issues arise when measuring OHRQoL in children due to their physical, cognitive, emotional, social, and language development phases because oral health and health cognition are age-dependent [7].

A myriad of age-specific instruments to quantity the impacts of oral problems on quality of life have been proposed [8]. OHRQoL is a multi-dimensional construct and a consensus on the number and nature of such dimensions has not been reached. Moreover, given the complexity of its multi-dimensionality and to enhance its applicability, simplifying the construct under the most relevant and meaningful dimensions to patients is essential for its practical and efficient use. Along these lines, *Oral Function*, *Orofacial Pain*, *Orofacial Appearance*, and *Psychosocial Impact* dimensions were identified as psychometrically sound [9–12] and clinically intuitive OHRQoL dimensions. While results were based on data for adult patients, it could be assumed that the impact of each oral condition could be measured with these four dimensions (4D) across all dental patients' age groups.

Given pediatric dental patients belong to an age group with specific clinical, behavioral, care-related, organizational, administrative, and legal characteristics [13], it would be relevant to investigate if these 4D are also the reasons for pediatric patients or their parents/guardians to visit dentists. Suppose OHRQoL dimensions are the primary areas where dental patients, regardless of their age, are impacted by oral disorders. In that case, this dimensional oral health impact should also be the reason why pediatric dental patients seek care.

In order to investigate this research question, dentists could be asked about their pediatric dental patients’ reasons for seeking oral health care and how these reasons align with the four OHRQoL dimensions. A study examining if the 4D are the underlying intention of pediatric patients to visit their dentists should be performed using a large number of international dentists with a broad range of clinical expertise and educational background, coming from dental settings across all six World Health Organization (WHO) regions. In a previous study, a systematic collection of such information determined 35 research studies, encompassing approximately 10,000 dental patients from the prosthodontic dental field and general population subjects from six countries, i.e., Croatia, Germany, Hungary, Japan, Slovenia, and Sweden [14]; the dimensions of the OHRQoL Project were identified out of the data mentioned above [14]. In hypothesis-generating [9], hypothesis-confirming [10], and validation analyses [12] the OHRQoL dimensions, i.e., *Oral Function, Orofacial Pain, Orofacial Appearance,* and *Psychosocial Impact*, were established, defined, proved to be valid, and generalizable to generic OHRQoL questionnaires or dental patient-reported outcome measures (dPROM) [11].

This study aimed to evaluate whether pediatric dental patients´ oral health concerns related to teeth, mouth, jaw fit into the 4D of the OHRQoL construct developed in the context of adults namely Oral Function, Orofacial Pain, Orofacial Appearance, and Psychosocial Impact based on their dentists' assessments.

## Methods

### Study participants

A convenience sample of international dentists from 32 countries was targeted in a web-based survey, representing all WHO regions with a minimum of three countries per WHO region. The African Region, Region of the Americas, South-East Asia Region, European Region, Eastern Mediterranean Region, and Western Pacific Region are the six world health regions as defined by the WHO. One reference dentist entitled as “center dentist” per country was selected and invited via e-mail by the authors (KRS or MTJ) to participate in this study. Center dentists were requested to invite at least ten other dentists from the country they came from to participate and complete the online English survey. Inclusion criteria were all active dentists with a valid dental license, who worked in public and/or private practice and/or in an educational institution, and who regularly diagnosed and treated dental patients in the last year, and had the capacity to read, understand, and respond to the English web-based survey.

In addition, we considered dentists that mentioned working only with pediatric dental patients, i.e., they diagnose and treat solely pediatric patients. In addition, we looked at dentists who diagnosed and treated pediatric dental patients besides regularly diagnosing and treating adult dental patients from different fields of dentistry.

The study was ethically approved by the Institutional Review Board (IRB) of the University of Minnesota, USA (IRB ID: STUDY00000864).

### Web-based survey

In this study, we assumed pediatric dental patients' subjective perspective of their oral health status is related to the multi-dimensional construct of OHRQoL, where 4D are embedded within the OHRQoL construct. Therefore, we collected data of current and future pediatric dental patients' oral health concerns, based on international dentists' reports, with an anonymous English-language web-based survey between June 2017 and July 2018. The survey was composed of three main questions with five response options per question. Responses were in terms of numbers or percentages so that the sum of all responses added up to 10 or 100, respectively. The questions were as follows:

1. Why did patients typically visit you when they had problems with their teeth (including dentures), mouth, or jaws?
  The patients visited me because of [% of patients]:
  Impaired oral function (eating, chewing, talking, etc.)
  Pain (dental, oral, facial, etc.)
  Impaired dental, oral, or facial appearance
  Broader psychosocial impacts/distress because of their oral health situation
  Other problems not mentioned above
2. To assess how your typical patients, match your most recent patients, please check the dental records or think of your last ten patients with oral health problems.
  How many patients came because of [number of patients]:
  Impaired oral function (eating, chewing, talking, etc.)
  Pain (dental, oral, facial, etc.)
  Impaired dental, oral, or facial appearance
  Broader psychosocial impacts/distress because of their oral health situation
  Other problems not mentioned above
3. You mentioned some patients visited you primarily for a preventative check-up. Why did they typically visit you when they came for a preventative check-up regarding their teeth (including dentures), mouth, or jaws?
  They visited me because they wanted to prevent [% of patients]:
  Impaired oral function (eating, chewing, talking, etc.)
  Pain (dental, oral, facial, etc.)
  Impaired dental, oral, or facial appearance
  Broader psychosocial impacts/distress because of their oral health situation
  Other problems not mentioned above

In instances where dentists responded in the form with "other problems not mentioned above," they were asked to write in a specifically marked field patient's other particular oral health problem or prevention need, which in their opinion does not fit into the four categories related to functional, painful, aesthetic, and psychosocial patients' impairment. A detailed analysis of dentists' responses related to "other problems not mentioned above" has been provided in the manuscript entitled “Why patients visit dentists—A study in all WHO regions.” [15].

Besides three key survey questions, dentists’ demographics and professional characteristics were collected, i.e., dentists’ country of practice, age, gender, dental field, the year since graduation from a school of dentistry, dental patients’ current oral health problems, patients’ referrals, and general (primary) dentists of their dental patients. We have selected six dental fields or specialties representing all major fields of dentistry across the world. The six fields were Restorative Dentistry (including Endodontics and Prosthodontics), Periodontics, Oral and Maxillofacial Surgery, Pediatric Dentistry, Orthodontics, and Oral Medicine and/or Temporomandibular Disorders (TMD). Dentists were asked to mark one or more dental fields of interest, i.e., the field(s) where they usually diagnose and treat their dental patients. Dentists who did not complete the entire survey were not included in the study analysis. The complete survey is available in the Additional file 1: web appendix.

### Analytical approach

The proportion of pediatric dental patients with four-dimensional (4D) oral health problems, i.e., problems related to teeth, mouth, jaws’ function, pain, appearance, or psychosocial impairments, was based out of the three main survey questions. The proportions of the aforementioned problems in the pediatric dental patient population and their parents or guardians who presented with current oral disorders and prevention were two principal outcomes of this study summated from questions one and three. The proportion of all pediatric dental patients' current 4D problems was extracted from the first survey question and applied for validation analysis.

In addition to the computation of proportions, we included dentists’ free-text responses, i.e., all patients’ oral health problems and prevention needs that were not categorized into the four OHRQoL dimensions and were described as “other problems not mentioned above.” Three study authors (MTJ, KRS, and SSe) independently screened all reported free-text and assessed them for their potentiality of classification into one of the four OHRQoL dimensions, i.e., *Oral Function, Orofacial Pain, Orofacial Appearance,* and *Psychosocial Impact*. After that, we included two determinants to assess pediatric dental patients, i.e., the six WHO regions and the six fields of dentistry.

For evaluation of pediatric dental patients’ current oral health problems, we conducted a multi-level mixed-effects logistic regression analysis (with two levels: patients and dentists) to model the binary effect variable, i.e., the presence ("1") or absence ("0") of 4D patients’ problems, with the assumption that dentist-level random outcomes considered the interdependencies among pediatric dental patients treated by the same dentists. For this purpose, we used Stata statistical software [STATA Release 14.2, rev.19; 2016, College Station, TX: StataCorp LP] with maximum likelihood estimation (MLE) and adaptive quadrature. On these grounds we assessed three models. The first model was a null model without predictor variables in the fixed part and a random variance component for dentists. The second and the third models were estimates with predictor variables for WHO regions or fields of dentistry in the fixed parts of the models (allowing the estimates to be adjusted for the geographical region where the dentist practiced and for the practice profile of rendered diagnoses and performed treatments) and a random variance component for dentists acquiring predicted probabilities of a positive response. An intraclass correlation coefficient (ICC) described the proportion of between cluster (dentist) variation in the total variation.

Pediatric dental patients’ oral health prevention needs were assessed with linear regression analysis by bootstrapping standard errors (SEs) in 1000 replications. Bootstrapping is a resampling or distribution-independent technique that approximates SEs based on the sample information [16]. Similar to previous analytical approaches, we assessed three models in which we included 407 dentists who treated pediatric dental patients for preventive oral health interventions. The first model was assessed without predictor variables, while the second and the third models were evaluated based on the six WHO regions and fields of dentistry used as indicator variables. In the analysis, including dentists who only treated pediatric dental patients, the WHO regions were extinguished to Europe versus non-Europe due to limited numbers of participating dentists for some non-European regions. In the validation analysis, we investigated how the study results of dentists’ 10 most recent pediatric dental patients can be generalized to the entire pediatric dental patient population. We calculated Pearson’s correlation coefficient, and based on Cohen’s *r* suggestions for its interpretation, an estimate ≥ 0.5 was considered a “large effect size.” Results are displayed in stacked bar charts and tables.

## Results

### Dentists’ general characteristics

In this international survey, 101 dentists from 23 countries who only treated pediatric dental patients were selected from a larger pool of 1580 dentists from 32 countries who participated in a larger web-based project [15]. Dentists had a mean age (SD) of 41.2 (9.3) years. More than three-quarters of them were females (78.2%). Approximately one-third of them (34.6%) practiced dentistry for ten or less years.

In addition, 523 dentists from 32 countries who responded to diagnose and treat pediatric dental patients besides regularly diagnosing and treating adult dental patients from different fields of dentistry were also selected. These dentists had a mean age (SD) of 38.2 (10.2) years. More than half of the participating dentists were females (53.7%). Approximately half of them (51.4%) practiced dentistry for ten or less years. A total of 415 dentists reported they were the primary (general) dentists of their dental patients, and for 127 dentists, their patients were not referred to them for dental treatment. One hundred sixteen dentists, i.e., 22.1%, answered that their patients visited them only when they were experiencing oral or orofacial problems. Study participants' characteristics are displayed in Table 1.

**Table 1:** General characteristics of 101 dentists who only treated pediatric patients and of 523 participating dentists who treated both pediatric and adult patients

Characteristics		Dentists who only treated pediatric patients (N = 101)		Dentists who treated pediatric and adult patients (N = 523)
		N (%) or Mean (SD)		
Age [years]		41.2 (9.3)		38.2 (10.2)
Gender [female]		79 (78.2)		337 (64.4)
*Years since graduation*				
0–10 years		35 (34.6)		269 (51.4)
11–20 years		38 (37.6)		156 (29.8)
21 or more years		28 (27.7)		98 (18.7)
Primary (general) dentist		60 (59.4)		415 (79.3)
Patients current oral health problems		16 (15.8)		116 (22.1)
Dental patients’ referrals		43 (42.5)		127 (24.2)
*Field of dentistry*^*#*^				
Restorative Dentistry		–		384 (73.4)
Periodontics		–		291 (55.6)
Oral & Maxillofacial Surgery		–		170 (32.5)
Pediatric Dentistry		101 (100)		523 (100)
Orthodontics		–		113 (21.6)
Oral Medicine and/or TMD		–		132 (25.2)
*WHO region*				
African Region		6 (5.9)		53 (10.1)
Region of the Americas		8 (7.9)		33 (6.3)
South-East Region		4 (3.9)		39 (7.4)
European Region		66 (65.3)		327 (62.5)
Eastern Mediterranean Region		12 (11.8)		42 (8.0)
Western Pacific Region		5 (4.9)		29 (5.5)
*Country*				
Australia		–		12 (2.2)
Austria		26 (25.7)		75 (14.3)
Bosnia & Herzegovina		1 (0.9)		9 (1.7)
Brazil		–		6 (1.1)
Chile		6 (5.9)		16 (3.0)
China		1 (0.9)		9 (1.7)
Colombia		1 (0.9)		3 (0.5)
Croatia		2 (1.9)		13 (2.4)
Denmark		1 (0.9)		6 (1.1)
Germany		–		28 (5.3)
Ghana		–		22 (4.2)
India		2 (1.9)		19 (3.6)
Iran		1 (0.9)		5 (0.9)
Italy		32 (2.9)		41 (7.8)
Japan		–		2 (0.3)
Northern Macedonia		4 (3.9)		25 (4.7)
Morocco		1 (0.9)		1 (0.1)
Nigeria		6 (5.9)		26 (4.9)
Panama		–		5 (0.9)
Russia		1 (0.9)		3 (0.5)
Saudi Arabia		8 (7.9)		31 (5.9)
Serbia		4 (3.9)		14 (2.6)
Singapore		4 (3.90)		6 (1.1)
Slovenia		18 (17.8)		82 (15.6)
South Africa		–		5 (0.9)
Sri Lanka		1 (0.9)		15 (2.8)
Sudan		2 (1.9)		5 (0.9)
Sweden		6 (5.9)		17 (3.2)
Switzerland		–		13 (2.4)
Thailand		1 (0.9)		5 (0.9)
United Kingdom		–		3 (0.5)
United States of America		1 (0.9)		1 (0.1)

*TMD* Temporomandibular Disorders, *WHO* World Health Organization

Dentists could mark one or more fields of dentistry

Dentists were frequently active in multiple fields of dentistry. Almost three-quarters of the participants (73.4%) generally diagnosed and treated patients in the field of restorative dentistry. The European Region was characterized by the most significant number of participating dentists, i.e., 62.5% of all dentists. Approximately 49 dentists per country completed the survey, and more than 100 dentists participated from four countries, i.e., Austria, Italy, Saudi Arabia, and Slovenia. For the 101 dentists who only diagnosed and treated pediatric dental patients, dentists’ characterization was similar to other dentists, who also treated adult patients beside pediatric patients (Table 1).

### Pediatric dental patients’ current oral health problems

**Fig. 1:**
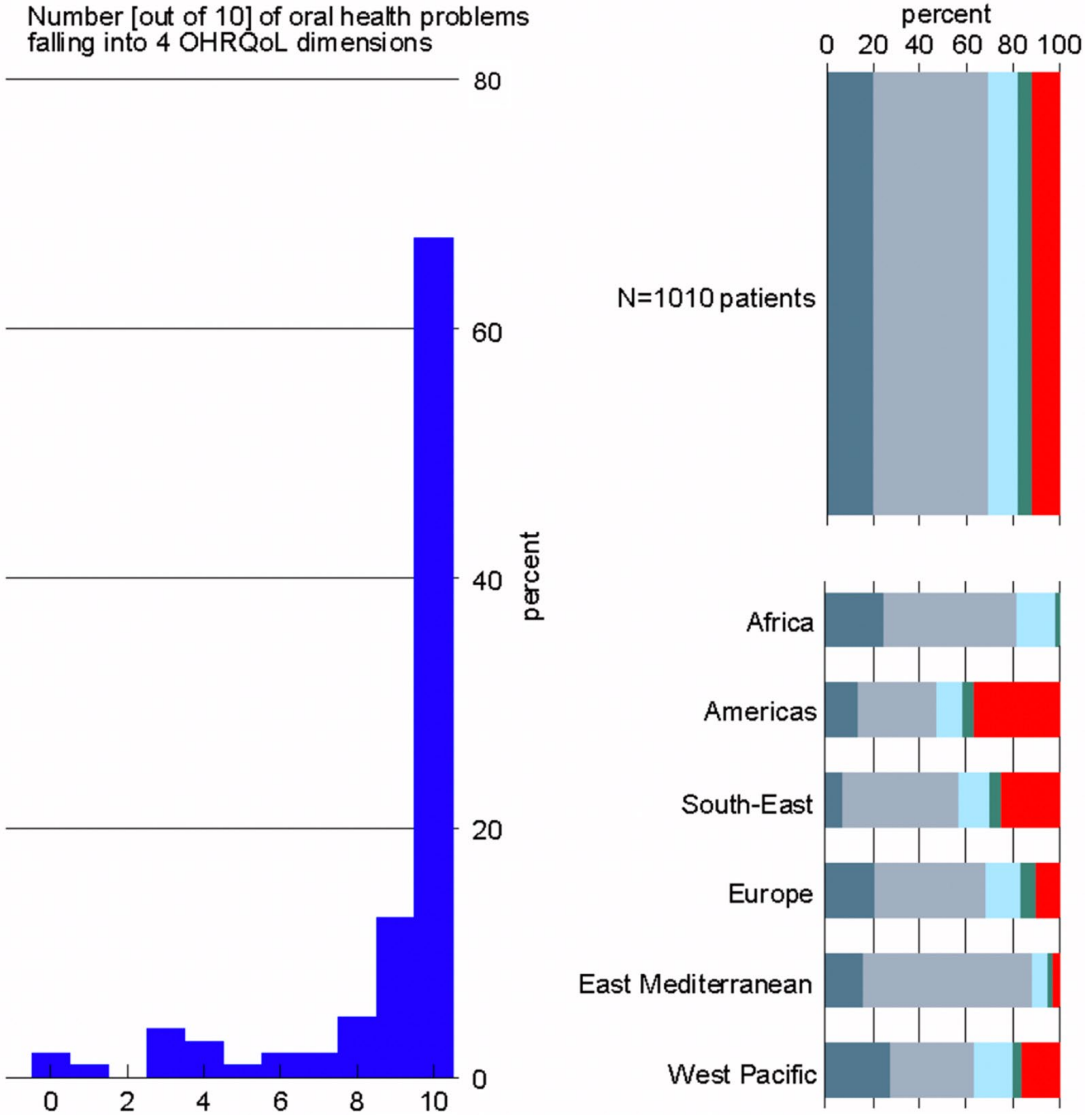
Number of pediatric patients [out of 10] per dental practice with a current four-dimensional oral health problem [left panel] and distribution of functional [Ash filled square], painful [white filled square], aesthetic [light blue filled square], psychosocial [green filled square], and other problems [red filled square] based on 1010 patients stratified by World Health Organization regions [right panel]

The majority of the 101 dentists who only diagnosed and treated pediatric dental patients (67%, n = 68) stated that their patients´ oral health disorders were associated with teeth, mouth, jaws’ function, pain, appearance, or psychosocial impact (Fig. 1, left panel).

Among 1010 pediatric dental patients with current oral or orofacial disorders, who were assessed by 101 dentists who only treated pediatric patients, 89% (n = 893) of the dental patients were impacted by 4D problems (Fig. 1, right panel). Considering patients were clustered within dentists, the model-derived probability of a 4D oral health problem was 90.0% in multi-level mixed-effect logistic regression (Table 2). The ICC was 0.79, i.e., more than three-quarters of the total variation of the outcome was due to variation between dentists’ responses. When adjusted for WHO regions, the proportion only slightly changed. When free-text responses were analyzed to derive corrected model-derived findings, the proportion increased to 96.0%. When we assessed 523 dentists who treated both pediatric and adult dental patients, previous proportions increased by 2.0% through 5.0%.

**Table 2:** Proportions of four-dimensional oral health problems based on dentists' reports who treated pediatric dental patients only and dentists' who treated both pediatric and adult dental patients

	Current 4D problems	
	Dentists who treated only pediatric patients (1010 patients)	Dentists who treated pediatric and adult patients (5230 patients)
Analysis	%	
Raw proportion	89.0	93.0
Model-derived proportion	90.0	94.0
Adjusted model-derived proportion	87.0–91.0	90.0–99.0
Corrected model-derived proportion	96.0	98.0

According to Cohen, the degree of correlation among the ten last pediatric dental patients’ and all dental patients’ oral disorders associated with teeth, mouth, jaw function, pain, appearance, or psychosocial impact, were “large” (range: 0.57–0.73).

### Proportions of pediatric dental patients´ preventive oral health concerns

**Fig. 2:**
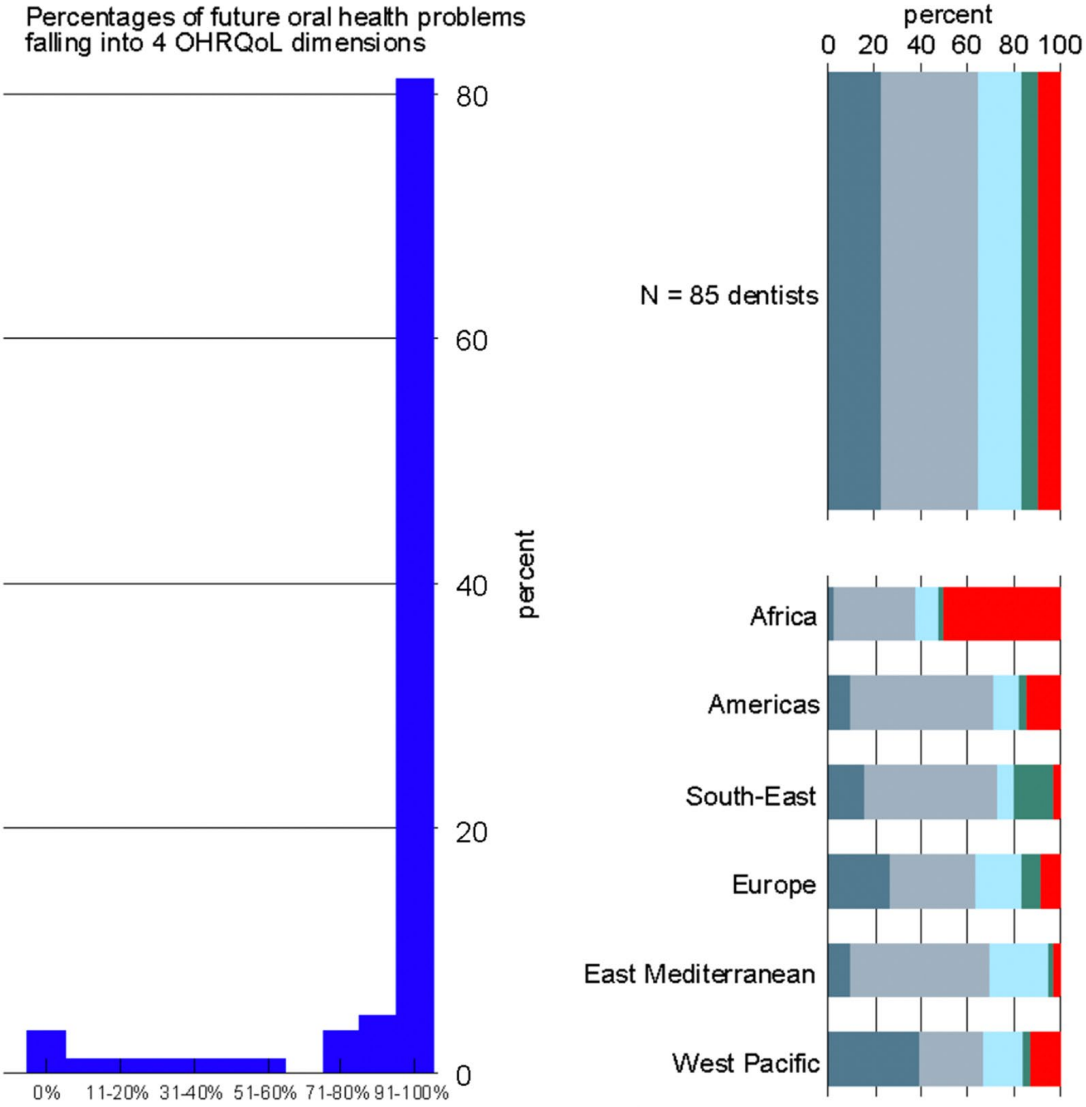
Eleven proportion brackets of patients intending to prevent four-dimensional oral health problems among all preventive patients per dental practice [left panel]. Distribution of problems related to functional [Ash filled square], painful [white filled square], aesthetic [light blue filled square], psychosocial [green filled square], and other problems [red filled square] patients wanted to prevent based on 85 dentists who only treated pediatric patients stratified by World Health Organization regions [right panel]

A vast majority (N = 85) of the 101 dentists who only treated pediatric dental patients implemented prevention oral health care among their dental patients. Between these dentists, more than 81.0% responded that all their patients’ future oral health problems were related to teeth, mouth, jaw function, pain, appearance, or psychosocial concerns (Fig. 2).

The proportion of 4D problems intended to prevent was 91.0% in the multi-level mixed-effect logistic regression (Table 3). When adjusted for the six WHO regions, proportions changed only slightly. When free-text responses of “other problems not mentioned above” were analyzed, the proportion increased to 96.0%. Compared to the dentists who treated pediatric and adult dental patients, the numbers decreased by 3.0 through 8.0%.

**Table 3:** Proportions of four-dimensional oral health problems intended to prevent based on dentists' reports who treated only pediatric patients and dentists who treated both pediatric and adult patients

	Current 4D problems intended to prevent	
	Dentists who treated only pediatric patients (N = 85)	Dentists who treated pediatric and adult patients (N = 407)
Analysis	%	
Raw proportion	91.0	94.0
Adjusted model-derived proportion	89.0–91.0	92.0–99.0
Corrected model-derived proportion	96.0	99.0

## Discussion

### Summary of study findings

This international survey performed in all WHO regions, showed that pediatric dental patients' oral health problems fit into the four OHRQoL dimensions, i.e., *Oral Function, Orofacial Pain, Orofacial Appearance,* and *Psychosocial Impact*, regardless of wether pediatric patients and their parents or guardians visited their dentist due to a check-up or because of the current oral health discomfort of the child.

### Pediatric dental patients’ oral health problems equivalence with the four OHRQoL dimensions

We engaged international dentists from 32 countries representing the six WHO regions to report their pediatric dental patients’ oral health problems. This study utilized a novel approach to study the pediatric dental patients' four OHRQoL dimensions in an international electronic survey to examine this research question. International surveys have already been used in other studies to present views related to themes such as the International Classification of Functioning, Disability, and Health [17] or its Health Children and Youth version (ICF-CY) [18]. Moreover, organized groups such as the World Dental Federation (FDI) regularly use international dentists’ surveys and questionnaires [19]. Recently, our international study group used data from the same web-based survey project and utilized similar methodological approaches for evaluating the fit of dental patients’ oral health problems within the four OHRQoL dimensions [15, 20]. We have found comparable results to this study, i.e., more than 90% of all adult dental patients’ current and future oral health problems coincided with Oral Function, Orofacial Pain and Appearance, and Psychosocial Impact dimensions of the OHRQoL construct.

Since our research question was unique and comprised the evaluation of only pediatric dental patients, the broader comparability of our findings is limited. Nevertheless, asking experts to assign items, in our case pediatric patients' oral health problems, into the four OHRQoL dimensions has been used many times. For example, Slade and Spencer initially grouped Oral Health Impact Profile (OHIP-49) items into seven conceptual domains, namely *Functional Limitation, Physical Pain, Psychological Discomfort, Physical Disability, Psychological Disability, Social Disability,* and *Handicap* [21]. Subsequently, it was shown by other investigators that only 4D are necessary when relating OHIP items to the initial domains [22]. Stable item (problem)-to-dimension distribution could be presented in a test re-test part in this study, demonstrating that dentists were able to agree on assigning items/problems to dimensions [22]. Typically, the consistency of such item/problem to dimension assignments is not studied. For instance, when dPROMs were thoroughly examined in two separate systematic reviews [23, 24], it was found that the vast majority of dPROMs’ authors appointed items to conceptual domains/dimensions without performing appropriate statistical analyses, only using their knowledge and intuition.

### The four OHRQoL dimensions as a universal set of dental patients’ oral health concerns

Our results i.e., that the 4D can be seen as a framework for pediatric dental patients' oral health problems and prevention needs are not unanticipated as these 4D were previously confirmed when OHIP-49 was examined [9–11]. The OHIP questionnaire contains a considerable number of OHRQoL items, which allows a thorough characterization of dental patients’ oral health problems when assessing adult dental patients but also pediatric dental patients.

Although OHIP the most commonly used psychometrically sound and valid questionnaire in OHRQoL studies in the pediatric dental patient population [25, 26], it should be noted that several dPROMs have been developed to specifically assess the self-report of pediatric dental patients’ OHRQoL outcomes. The most often used ones are the Child Perceptions Questionnaire (CPQ) [27], the Child Oral Health Impact Profile (COHIP) [28], the Child Oral Impacts on Daily Performances (C-OIDP) [29], and the Early Childhood Oral Health Impact Scale (ECOHIS) [30]. Although all these pediatric dPROMs incorporate the well-known multi-dimensionality of OHRQoL, there is no agreement with the number and nature of incorporated dimensions.

So far, no studies of these pediatric dPROMs exist that would support the hypothesis that the 4D fit for pediatric dental patients. For example, the CPQ was developed to assess the OHRQoL of pediatric dental patients (children and adolescents) aged between 8 and 14 years. It assesses their personal experience through four conceptual domains related to oral impairments, i.e., *Oral Symptoms, Functional Restrictions, Emotional Impairment,* and *Social Impairment*. These domains could be well assigned to the four OHRQoL dimensions only, e.g., the *Socio-Emotional* dimension represents well the *Psychosocial Impact* and the *Orofacial Appearance* dimensions of the 4D OHRQoL construct. In contrast, the *Symptoms-Functioning* dimension represents *Oral Function* and *Orofacial Pain* dimensions appropriately [31].

Moreover, it has been demonstrated that OHIP and CPQ showed similar results when applied in the same patient population group [32]. A recent systematic review by Ferrando-Magraner et al. [32] compared OHIP and CPQ regarding patients’ malocclusions and revealed comparable results in administration of both dPROMs for pediatric dental patients. The two dPROMs, i.e., OHIP and CPQ, reported both significant improvement in OHRQoL after orthodontic treatments [32]. All these findings indicate OHRQoL assessment in pediatric dental patients, i.e., for both children and adolescents, share many similarities with OHRQoL assessment in adults, suggesting oral health impacts are comparable across the entire life span.

### Strengths and limitations

Our study had several strengths. We included 32 countries from all six WHO regions, with each region being represented by at least three countries. Among these 32 countries, the seven worldwide largest countries, except for Canada, were included. We had a large sample size of international dentists. Although the number of dentists who only treated children was not as large and these dentists were not equally distributed across WHO regions. Findings for this specific group were similar to that of dentists who treated all ages (children and adults) as well as for dentists who only treated adults. Furthermore, we included dentists who work in all dental settings, i.e., dentists working in public or private practices and dentists working in educational institutions. Due to the inclusion of patients from different backgrounds, we have captured the entire spectrum of dental patients living in all six WHO regions.

This study also had limitations. We were not able to calculate a response rate due to the anonymity of data collection. Furthermore, there was an unequal distribution of pediatric dentists across WHO regions. However, analyses focusing on the geographical region where the dentist practiced showed that only slight differences compared to analyses not including the geographical region, indicating that the dentist's place did not notably influence pediatric dental patients’ oral health coverage. Also, our participating dentists needed to be fluent in English. A particular dentist recruited other dentists within the same country. Lastly, the study's inclusion criteria and recruitment process made participating dentists a convenience sample of all the country's dentists that are not representative of a country's dental workforce.

### Outcomes

*Oral Function*, *Orofacial Pain*, *Orofacial Appearance*, and *Psychosocial Impact* dimensions of the OHRQoL construct provide an intuitive and pragmatic framework for dental practice and research settings worldwide, not only for adult dental patients but for pediatric dental patients as well.

If these 4D were assessed in OHRQoL measurements in dental patients of all ages, a unified measurement system would enable precise measurement of the impact of oral disorders across the entire population. The change in four dPROM scores would represent the patient-perceived dental treatment effects. Studies can then be designed to investigate which treatments are best for patients, and dentists could apply this knowledge in practice, i.e., an evidence-based dental practice could be performed. While the importance of 4D dPROMs in dental practices has been emphasized for dental fields, e.g., prosthodontics [7], orthodontics [33] or care delivery approaches such as value-based dental care [34], dental disciplines that treat only or mostly children (pediatric dentistry or orthodontics) would benefit even more from the 4D approach to outcome measurement. Pediatric dentistry and orthodontics’ intervention effects are assumed to be substantial and to last a long time, conceptually even changing the oral health trajectory for the patients over the entire life. Only an outcome measurement system that starts in the pediatric age range but crosses over to adults would be able to identify and confirm these effects.

## Conclusions

The four fundamental components of a dental patient's oral health experience, i.e., the four OHRQoL dimensions (Oral Function, Orofacial Pain, Orofacial Appearance, and Psychosocial Impact), are also applicable for the pediatric patient population. They apply regardless of whether pediatric patients have current or will have future oral health concerns. These dimensions should therefore be considered when measuring OHRQoL in this patient population in future studies.

## Supplementary Information

**Additional file 1.** Web appendix.

## Data Availability

The datasets used and/or analysed during the current study are available from the [MTJ] on reasonable request.

## Abbreviations

OHRQoL: Oral health-related quality of life
WHO: World Health Organization
4D: Four-dimensional
ICC: Intraclass correlation coefficient
TMD: Temporomandibular disorders
OHIP: Oral health impact profile
dPRO: Dental patient-reported outcome
dPROM: Dental patient-reported outcome measure
