# Supplementary material for: Pediatric patients’ reasons for visiting dentists in all WHO regions
Source: Health Qual Life Outcomes. 2021 Jun 13;19:165. doi: 10.1186/s12955-021-01801-0 (PMC8201707; doi:10.1186/s12955-021-01801-0)
Supplement: Supplementary file 1 — Additional file 1. Web appendix. [file 12955_2021_1801_MOESM1_ESM.pdf]

International Dentists' Survey

Welcome

Dear Colleague,

We, an international research team led by Dr. Mike T. John from the University of Minnesota, USA and Dr. Ksenija Rener-Sitar from the University of Ljubljana, Slovenia, are performing an international survey in all world regions specified by the WHO. We are studying the main reasons why patients visit their dentists.

The survey is anonymous and takes 5 to 10 minutes to complete. We kindly invite you to participate.

For the purpose of this survey, we would like you to divide patients who visit you into two groups:
1. Patients with one or more current oral/orofacial health problems. These patients have an oral/orofacial health condition that affects them and they perceive this impact, e.g., tooth pain.
2. Patients without a current oral/orofacial health problem. These patients visit you to prevent future problems. They typically come for a regular check-up, a consultation, a second-opinion, or for a dental hygiene appointment. Sometimes, dentists refer asymptomatic patients to you, e.g., patients with chronic periodontal disease, in order to prevent oral health problems for them. These patients are also included in this group.

When patients (e.g., children or patients with disabilities) are accompanied by a caregiver, parent, or guardian, please consider them together. For example, children may not have a good understanding of potential oral health problems, but their parents brought them in because they would like to prevent them for their kids.

If you are ready to participate, please click “**Next**”.

Survey questions

1. Why did patients typically visit you when they had problems with their teeth (including dentures), mouth, or jaws?
Please only consider the patients' primary problem!

**The patients visited me because of:**	**% of patients:**
**- Impaired oral function (eating, chewing, talking etc.)**	0
**- Pain (dental, oral, facial etc.)**	0

**- Impaired dental, oral, or facial appearance**	0
**- Broader psychosocial impacts/distress because of their oral health situation**	0
**- Other problems not mentioned above**	0
**Total**	0

1.1. You mentioned that some patients had "...other problems not mentioned above".
Please write down the most important problem, which doesn't fit into any of the four listed categories *(Function, Pain, Appearance, Psychosocial Impact).*

2. To assess how your typical patients match your most recent patients, please check the dental records or think of your last 10 patients with oral health problems.
Please only consider the patients' primary problem!

**How many patients came because of:**	**No. of patients:**
**- Impaired oral function (eating, chewing, talking etc.)**	0
**- Pain (dental, oral, facial etc.)**	0
**- Impaired dental, oral, or facial appearance**	0
**- Broader psychosocial impacts/distress because of their oral health situation**	0
**- Other problems not mentioned above**	0
**Total**	0

2.1. You indicated that some of your last 10 patients had "...other problems not mentioned above".
Please write down the most important problem, which doesn't fit into any of the four listed categories *(Function, Pain, Appearance, Psychosocial Impact)*.

3. Did any patients visit you primarily for a preventative check-up regarding their teeth (including dentures), mouth, or jaws?

- ○ **Yes, they did.**
- ○ **No, they visited me only when they had primarily problems with their teeth (including dentures), mouth, or jaws.**

3.1. You mentioned that some patients visited you primarily for a preventative check-up. Why did they typically visit you when they came for a preventative check-up regarding their teeth (including dentures), mouth, or jaws?
Please only consider the patients' primary intention!

**They visited me because they wanted to prevent:**	**% of patients:**
**- Impaired oral function (eating, chewing, talking etc.)**	0
**- Pain (dental, oral, facial etc.)**	0
**- Impaired dental, oral, or facial appearance**	0
**- Broader psychosocial impacts/distress because of their oral health situation**	0
**- Other intentions not mentioned above**	0
**Total**	0

3.2. You wrote that some patients came because they wanted to prevent "...other problems not mentioned above".
Please write down the most important intention, which doesn't fit into any of the four listed categories *(Function, Pain, Appearance, Psychosocial Impact)*.

4. Please choose one global assessment to describe how well the four problem categories *(Function, Pain, Appearance, Psychosocial Impact)* fit your patients' current and future oral health concerns.

**Poor fit**	**Fair fit**	**Good fit**	**Very good fit**	**Excellent fit**
○	○	○	○	○

Demographics

5. To characterize the group of participating dentists, please also provide the following information:

5.1. Country where you currently work as a dentist:

5.1.1. Please write which "other country" you live in.

5.2. Year of graduation from school of dentistry:

5.3. Your age:

5.4. Your gender:

- **Female**
- **Male**

5.5. Do the majority (> 50%) of your patients see you because of current problems? (as compared to patients coming for a check-up)

- ○ **Yes**
- ○ **No**

5.6. Are the majority (> 50%) of your patients referred to you by other dentists?

- ○ **Yes**
- ○ **No**

5.7. Are you the general (i.e., primary) dentist for the majority (> 50%) of your patients?

- ○ **Yes**
- ○ **No**

5.8. In which major field(s) do you regularly diagnose and treat patients?
Please mark all areas that apply for you!

- ☐ **Restorative Dentistry (including Endodontics and Prosthodontics)**
- ☐ **Periodontics**
- ☐ **Oral and/or Maxillofacial Surgery**
- ☐ **Pediatric Dentistry**
- ☐ **Orthodontics**
- ☐ **Oral Medicine and/or Temporomandibular Disorders**
